# Single-stage in situ suture repair of multiple-ligament knee injury: a retrospective study of 17 patients (18 knees)

**DOI:** 10.1186/s12891-016-0894-1

**Published:** 2016-01-22

**Authors:** Xingyi Hua, Hui Tao, Wang Fang, Jian Tang

**Affiliations:** Department of Orthopaedic Surgery, the First Affiliated Hospital of Anhui Medical University, No.218 Ji-xi Road,Hefei, Anhui, 230022 People’s Republic of China

**Keywords:** Knee dislocation, Multiple-ligament injury, In situ repair, Single stage

## Abstract

**Background:**

Multiple-ligament injured knee (MLIK) is a rare but severe injury. Although the principles of MLIK management have progressed over the past 40 years, there is a paucity of high-quality evidence upon which to base the management of MLIK. Treatment strategies for MLIK are challenging for most orthopedic surgeons, and the optimal treatment remains controversial, especially with regard to repair vs. reconstruction of the ligaments. The aim of the present study was to observe clinical outcomes of single-stage in situ suture repair of knee dislocation with multiple-ligament injury using nonabsorbable suture material.

**Methods:**

Consecutive patients with MLIK between 2002 and 2010 were included, for a total of 25 patients with knee dislocation. 17 patients (18 knees) with closed knee dislocation with a mean follow-up of 4.8 ± 1.3 years were retrospective analyzed. All patients were treated surgically with single-stage in situ suture repair for all injured ligaments and followed a standardized postoperative rehabilitation protocol. The VAS score, satisfactory score, total SF-36 score, Lysholm score, Tegner score, the Meyers functional rating and the ranges of motion and knee stability were used to evaluate outcomes.

**Results:**

At final follow-up, mean visual analog scale score was 2.4 ± 0.9, patient satisfaction score was 8.0 ± 1.1, 36-item Short-Form Health Survey total score was 85.5 ± 10.4, and mean Lysholm score was 87.5 ± 7.7. There were significant differences between mean preinjury and postoperative Tegner activity scores (5.6 ± 1.4 and 3.4 ± 1.7, respectively; *P* < 0.01) and in mean range of motion between the injured and contralateral knees (112.5 ± 8.4° and 129.6 ± 10.3°, respectively; *P* < 0.01). At final follow-up, no patient demonstrated obvious ligamentous laxity, and only one patient was unable to return to work. Three patients had knee joint stiffness, two had wound problems (infection or fat liquefaction), and two had heterotopic bone formation.

**Conclusions:**

Single-stage in situ suture repair of injured ligaments confers advantages of reliable fixation and early exercise. It could be considered as an alternate and effective option in the dislocation knee with multiple-ligament injury.

## Background

Multiple-ligament injured knee (MLIK), defined as an injury involving at least three of the four main ligaments of the knee [1], is a rare but severe injury. It always presents as knee dislocation. The incidence has been reported to be only 0.001 to 0.013 % of all emergency department injuries [2–4]. However, because of spontaneous reduction and missed diagnosis, the actual incidence of knee dislocation may be slightly higher [5, 6].

Although the principles of MLIK management have progressed over the past 40 years, its optimal treatment is debated [1]. There is a paucity of high-quality evidence upon which to base the management of MLIK, and treatment strategies for MLIK are challenging for most orthopedic surgeons. Because nonoperative management of MLIK generally leads to poor short- and long-term outcomes, most orthopedic surgeons prefer to treat MLIK surgically [7–10]. However, surgical techniques vary and are controversial, especially with regard to repair vs. reconstruction of the ligaments [9, 11–15]. One reason for this is the lack of agreement among studies; several authors have demonstrated the failure rate of repair to be markedly higher than that of reconstruction [11, 12], while Owens et al. [13] demonstrated the failure of early repair that was not coupled with a modern rehabilitation program. Therefore, the purpose of the present study was to retrospectively analyze the clinical outcomes of patients with MLIK treated with single-stage in situ suture repair followed by a standardized postoperative rehabilitation protocol.

## Methods

### Patients

Using a standardized protocol, a retrospective review of our patient databases was undertaken. This yielded 25 consecutive patients with knee dislocation who underwent surgical treatment by a single senior surgeon between January 2002 and October 2010. This study was approved by the ethics committee of the first affiliated hospital of Anhui Medical University, and the informed consents were obtained from all the participants. The diagnosis of knee dislocation was made on the basis of clinical signs and symptoms and magnetic resonance imaging. Exclusion criteria were as follows: open trauma, severe cranial or cerebral injury, vascular injury requiring emergency vascular surgery, associated fractures requiring external fixation, or initial treatment performed at another institution. Ultimately, 19 patients with 20 MLIKs were included in the present study, and all patients were treated surgically with single-stage in situ suture repair.

### Surgical technique

**Fig. 1:**
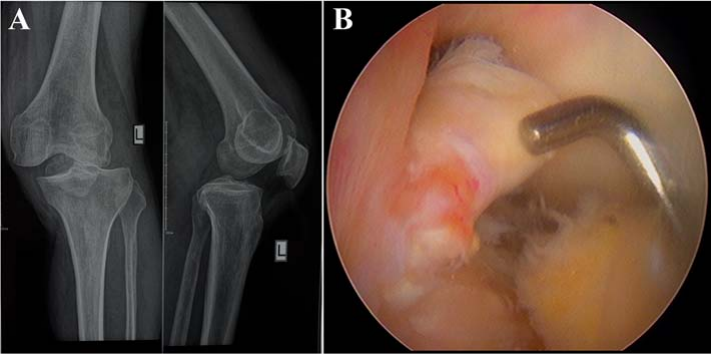
**a** X-ray showed the left knee dislocation; **b** Arthroscopy showed the anterior cruciate ligament was rupture completely through its middle (as showed by pin)

All operations were performed 5–10 days after injury. The patient was placed under general anesthesia and positioned supine on the operating table. The uninjured leg was extended, and the hip and knee on the injured side were flexed to 90° and the lateral thigh supported by a solid baffle. General anesthesia with controlled hypotension was used, and no tourniquet was placed. Physical examination and routine arthroscopy were first performed to identify the ligaments injured (Fig. 1). The anterior cruciate ligament (ACL), posterior cruciate ligament (PCL), posterolateral corner (PLC), and medial collateral ligament (MCL) were repaired using the following open surgical technique.

**Fig. 2:**
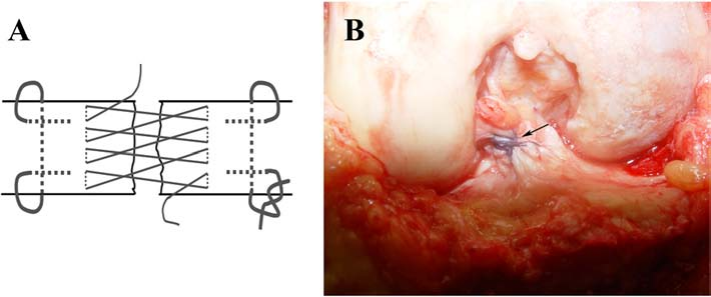
**a** Schematic of running baseball cross-stitch; **b** Anterior cruciate ligament repaired in situ by using running baseball cross-stitch (*black arrow*)

After sterile preparation and draping, an anterior incision was created slightly off the midline to allow for ACL and PCL tunnel placement. The articular space was opened to remove blood clots, and the stumps of ACL and PCL were exposed. The anteromedial and posterolateral bundles of the ACL and PCL were carefully identified. They were then repaired using a running baseball cross stitch (Fig. 2) with 2-0 nonabsorbable COBRAID™ suture (Smith & Nephew, Inc., Andover, MA, USA) for repair. With the knee tightly flexed, an Arthrex guide (Arthrex, Inc., Naples, FL, USA) was used to create the four femoral lead tunnels of the ACL at the center of the two bundles for reattachment of the ACL to the medial wall of the femoral condyle, drilling from anteromedial to posterolateral, and ensuring that the bone bridge between the two tunnels was about 1 cm. The femoral leads tunnels for the PCL, tibial head tunnels for the ACL, and tibial leads tunnels for the PCL were created using the same technique. During preparation of the tibial leads tunnels for the PCL, when the Kirschner wire crossed the posterior tibial cortex, extreme care was taken to avoid injuries to nerves and blood vessels. Next, a suture passer was used to guide the suture lines through each tunnel, simultaneously tightening the ends of the suture lines of the avulsed bundle stump, and then knotting each suture line outside the bone tunnel on the corresponding bone bridge. This completed reattachment of the avulsion stump in situ. Meniscal damage was repaired using sutures or by trimming, depending on the site of the injury.

Next, The PLC was approached via a posterolateral incision, being careful to maintain a 6- to 8-cm skin bridge between the two incisions. The popliteus, popliteofibular ligament, capsule, lateral collateral ligament, iliotibial band, and the biceps femoris were repaired to the femoral epicondyle, fibular head, or the lateral tibia, depending on the site of detachment. After decorticating the bone at the site of insertion, two to five nonabsorbable suture anchors were applied using cross-stitch technique. Complete avulsion of the MCL was repaired through the midline incision using a similar method, and partial injuries were not treated surgically. Before the wound was closed, the knee was brought through a 90-degree range of motion. Anterior and posterior drawer tests and the Lachman test were gently performed to verify stability.

### Rehabilitation protocol

There are few reports on postoperative rehabilitation after open repair of MLIK. A hinged knee brace locked in 30° of flexion was used to protect the stability of the injured knee, and a standard rehabilitation protocol was subsequently performed. Patients were allowed to perform quadriceps isometric exercise and straight-leg raise on postoperative day 1. Care was taken to avoid varus and valgus stress in patients who had undergone PLC and/or MCL repairs. Patients began physical therapy 1 week later at our institution on an outpatient basis. The brace could be unlocked and the knee was brought through a full range of motion as tolerated. After 4 weeks, nonweightbearing activities and passive knee-flexion and -extension exercises were begun, gradually increasing the range of flexion from 0 to 120°. The third month postoperatively, closed-chain exercise and hamstring co-contractions were initiated. At postoperative months 4–5, patients began open-chain exercises and walking, partial weightbearing with crutches, while gradually increasing the range of the motion. At 6 months, patients could partially resume daily activities and begin progressive resistive exercise. At the goal of 7 months postoperatively, patients could walk, bearing full weight without crutches.

### Follow-up evaluations

In all, 17 patients (18 knees) were followed for a mean of 4.8 ± 1.3 years (range, 2.4–7.3 years). Patients were examined by an independent senior orthopedic resident.

To assess clinical outcomes, we used a visual analog scale (VAS) score from 0 (no pain) to ten (worst pain) and a patient satisfaction score of 10 to 0, with a higher score indicating greater satisfaction. Self-administered questionnaires, including the Lysholm score [16], the Tegner score [17], and the 36-item Short-Form Health Survey® (SF-36) total score, were also used to evaluate clinical outcome. Finally, the Meyers functional rating was used to determine postoperative function [18, 19].

Range of motion and knee stability were also evaluated on physical examination. Range of motion was measured using standard goniometry; loss of flexion and extension were calculated by comparing the injured knee with the uninjured knee. The two patients with bilateral knee dislocation were excluded from this analysis of range of motion. To estimate the laxity of the ACL and PCL, a KT1000™ arthrometer (MEDmetric® Corp., San Diego, CA, USA) was used to perform Lachman and posterior drawer tests, respectively. Collateral ligament laxity was tested clinically by applying varus or valgus stress in extension and 30° of flexion. The stability of the PLC was tested using the Cooper asymmetry test (Dial test), which was performed in 30 and 90° of flexion.

Postoperative complications, including deep vein thrombosis (DVT), infection, suture granuloma, re-rupture, fibrosis, common peroneal nerve palsy, and heterotopic bone were recorded.

### Statistical analysis

Statistical analysis was performed using SPSS for Windows, Version 13.0 (SPSS Inc., Chicago, IL, USA). All data are presented as mean ± standard deviation. The results were analyzed using a Student’s *t*-test, and significance was defined as *P* < 0.05for 95 % confidence.

## Results

### Patients and epidemiological profiles

After applying exclusion criteria, seven patients were excluded, leaving 19 patients with 20 MLIKs. All underwent single-stage in situ repair. Two patients were lost to follow-up after the 3-month follow- up visit; one moved to a distant city and the other could not be contacted by telephone. Therefore, 17 patients (11 men, six women; mean age at the time of injury, 38.8 ± 11.3 years [range, 19–62 years]).with 18 MLIKs (89.5 % follow-up) were included in the present study. Details of patient demographics and injury patterns are shown in Table. 1.

**Table 1:** Patient demographics and injury characteristics

No.	Gender	Age	Mechanism	Follow-up	KD	ACL	PCL	MCL	LCL	PCL	Meniscus	Fracture	VAS	Satisfactory	Lysholm	Tegner score				Complication
				(years)									score	score	score	Pre	post	SF-36 score	motion	
Unilat																				
1	M	37	Vehicle	3.7	III-L	+	+		+	+			2.1	8.9	92	6	4	86	114°	
2	F	46	Vehicle	4.2	IV	+	+	+	+	+	+		1.9	9.2	93	4	2	91	109°	
3	F	23	Vehicle	6.2	III-M	+	+	+			+		1.2	9	95	6	3	95	116°	
4	M	29	Vehicle	5.1	V	+	+	+	+	+	+	+	2.7	7.2	93	7	6	91	111°	Fat liquefaction
5	F	56	Fall	4.9	IV	+	+	+	+	+	+		1.6	7.6	82	5	1	85	98°	Arthrofibrosis
6	M	19	Football	6.3	III-M	+	+	+	+		+		2.2	8.9	95	9	6	100	134°	
7	M	62	Hit	3	III-L	+	+		+	+	+		1	9	93	5	3	78	103°	Wound infection
8	M	37	Vehicle	4.9	III-L	+	+		+	+	+		2.5	8.7	93	6	5	91	122°	
9	F	31	Vehicle	5.2	V	+	+	+	+	+	+	+	4.2	5.1	71	5	1	57	109°	Heterotopic bone
10	M	42	Fall	7.3	IV	+	+	+	+	+	+		2.7	7.9	90	6	4	80	111°	Arthrofibrosis
11	M	29	Vehicle	2.4	III-L	+	+		+	+			1.8	8.6	94	7	5	89	120°	
12	F	34	Vehicle	5.7	IV	+	+	+	+	+	+		2.7	8.9	85	5	3	90	113°	
13	F	50	Hit	3.8	IV	+	+	+	+	+	+		2.6	7.2	81	3	1	84	104°	Arthrofibrosis
14	F	38	Hit	4.6	IV	+	+	+	+	+	+		1.9	8.5	90	4	4	92	107°	
15	M	44	Vehicle	5.5	IV	+	+	+	+	+	+		2.1	7.7	85	5	4	87	116°	
16	M	37	Vehicle	4	III-M	+	+	+			+		3.2	8.1	92	6	4	91	113°	
Bilat																				
17-Left	M	46	Vehicle	5.3	IV	+	+	+	+	+	+		3.1	7	80	6	1	67	104°	
17-Right	M	46	Vehicle	5.3	IV	+	+	+	+	+	+		4.3	6.2	71	6	1	67	92°	Heterotopic bone
Mean ± SD		38.8 ± 11.3		4.8 ± 1.3									2.43 ± 0.89	7.98 ± 1.12	87.50 ± 7.73	5.59 ± 1.37	3.35 ± 1.69	85.53±10.44	112.50 ± 8.44^a^	

^a^Data for patient No. 17 (bilateral MLIK) are excluded

All patients had closed injuries that were reduced in the emergency room. The ligamentous injuries were on the left in seven cases, on the right in nine cases, and bilateral in one case. The mechanism of the injury was a motor vehicle accident in 11 patients (12 knees), a direct hit in three patients, fall from a height in two patients, and from football in one patient. Patients were categorized by combinations of ligament injuries using the modified Schenck system, as follows: KD III-M, *n* = 3; KD III-L, *n* = 4; KD IV, *n* = 9; and KD V, *n* = 2.

Twelve patients (13 knees, 72.2 %) had at least partial injury of the common peroneal nerve. Seven (53.8 %) of these knees had partial sensory loss, five (38.5 %) had partial sensory and motor loss, and one (7.7 %) had complete sensory and motor loss. Associated meniscus tear was also common in our study group, and only two patients had significant fractures with ipsilateral tibial plateau fracture (one Schatzker type I and one Schatzker type III).

### Clinical outcomes

At final follow-up, mean VAS pain score was 2.4 ± 0.9 (range, 1.0–4.3); 15 patients (88.2 %) had a VAS pain score ≤3. Final patient satisfaction mean score was 8.0 ± 1.1 (range, 5.1–9.2), and only one patient had a satisfaction score <6. At final follow-up, mean total SF-36 score was 85.5 ± 10.4 (range, 57–100), and only one patient scored <60.

Mean postoperative Lysholm score was 87.5 ± 7.7 (range, 71–95). No patient scored below 60, and only one patient scored below 70. Mean preinjury Tegner activity score injury was 5.6 ± 1.4 (range, 3–9); postoperatively it decreased to 3.4 ± 1.7 (range, 1–6) demonstrating significant improvement (*P* < 0.01). The Meyers functional rating yielded one excellent, eight good, six fair, and two poor results. Of the 17 patients analyzed, 12 (70.6 %) were able to return to their previous work with little to no activity modification, four (23.5 %) were able to do light duty only, and only one patient (5.9 %), who suffered bilateral knee dislocation, was unable to return to work.

All patients underwent a follow-up physical examination to evaluate range of motion and ligamentous stability, and the results in detail are showed in Table. 2. Except the patients with bilateral knee dislocation, the total average arc of motion at the final follow-up was lower than uninjured knees (112.5° ± 8.4° (range, 98°–134°) vs. 129.6° ± 10.3° (range, 115°–141°)), and the difference was significant (*P* < 0.01). For flexion loss, two patients lost less than 5°of flexion, ten patients between 6°and 15°, three patients between 16°and 25°and only one patient more than 25°. Furthermore, there was no patient who had an anterior laxity or posterior laxity more than 3 mm at the final follow-up. Compared to the uninjured knees (exception bilateral injured knees), the mean cooper asymmetry testing of the injured knees at 30° decreased 0.8° ± 0.4° (range, 0°–1.2°) and at 90° decreased 0.5° ± 0.2° (range, 0°–0.7°).

**Table 2:** Outcome parameters

Outcome parameter		Mean ± SD	Range
Flexion loss		17.1° ± 8.6°	1°–29°
Extension loss		1.7° ± 2.2°	0°–5°
Anterior laxity		1.4 ± 0.9 mm	0–1.8 mm
Posterior laxity		0.8 ± 0.4 mm	0.1–1.2 mm
Varus stability	0°	0.1 ± 0.1 mm	0–0.2 mm
	30°	0.5 ± 0.2 mm	0–0.7 mm
Algus stability	0°	0.1 ± 0.1 mm	0–0.2 mm
	30°	0.5 ± 0.2 mm	0–0.7 mm
Cooper asymmetry testing	30°	5.4° ± 3.6°	0°–11°
	90°	3.7° ± 4.5°	0°–8°

### Complications

No DVT, compartment syndrome, iatrogenic neurovascular compromise, or deep infection was identified in our patients. Two patients experienced wound problems, including infection and fat liquefaction, which resolved after treatment with antibiotics and dressing changes. Three of the patients had arthrofibrosis: one received stereoarthrolysis under anesthesia 20 weeks postoperatively, and the other two received arthroscopic lysis of adhesions, one 33 weeks and the other 45 weeks postoperatively. Mild heterotopic bone formation developed in the knee of the patient with a Schatzker type III tibial plateau fracture and in the right knee of the patient with bilateral knee dislocations. Although the patient who suffered tibial plateau fracture had good knee joint activity, she suffered the pain around her knee joint, and the pain associated with climate change. It may be caused by the scar around her knee joint. The other patient had poor result might due to not well rehabilitation. As the patient suffered bilateral knee dislocations, he needed pay more attention to rehabilitation than other patients.

## Discussion

Because it is rare and difficult to treat, MLIK is particularly challenging for orthopedic surgeons. As the incidence of MLIK is rare low, treatment has relied heavily on case series, and is controversial [1, 20]. Therefore, the goal of the present study was to retrospectively analyze the 17 consecutive patients we treated with single-stage in situ suture repair. After a mean follow up of 4.8 years, we found that treatment with in situ suture repair provided the majority of patients with high patient satisfaction and a good functional result, with the Meyers functional rating demonstrating at least fair results in 15 patients (88.2 %) and poor results in only two. Although many of the patients had slight postoperative symptoms or/and functional limitations subjectively, 16 patients (94.1 %) could return to sports activities or daily activities.

Over a long-term study, conservative treatment for MLIK is no longer recommended, but multiple operative strategies have been performed. These include procedures performed in one or two stage [21–23], early or late post injury [24, 25], open or arthroscopically [6, 22, 26], and with repair or reconstruction [12, 13, 20, 27]. Two-stage management of MLIK, which involves suturing of the MCL and/or LCL within 8–10 days of injury followed by reconstruction of the ACL and/or PCL after 6–8 weeks, is widely accepted [28, 29]. However, a two-stage surgical strategy requires patients to undergo operation and anesthesia twice, which can increase risk, discomfort, and medical costs.

Three weeks after injury is often defined as the threshold between *acute* and *chronic* injury. After 3 weeks, the ligament stumps tend to scar, retract, and form granulation tissue, and suture repair of the rupture becomes difficult [4, 24, 30, 31]. In addition, long-term rehabilitation of an injured knee for delay treatment is prone to arthrofibrosis. Harner et al. [31] reported that patients treated in the acute period had better Knee Outcome Survey Sports Activity, Daily Living, and Lysholm scores than patients treated in the chronic phase. Moreover, Richter et al. [11] showed significantly better results for ligamentous suture repair performed within 1 week of injury than for delayed repair (>1 week). Therefore, suture repairs for MLIKs should be performed within 1 week of, and not more than 3 weeks after, injury. MLIK is a serious trauma and is always associated with torn capsular tissue, and the risk of compartment syndrome due to fluid leakage increases when patients are treated arthroscopically early after injury. Therefore, all the patients in the present study were treated with open surgery in a single stage within 5–10 days of injury.

Several studies have reported that the knee ligaments, especially the ACL, PCL, and PLC, were not suitable for primary repair, and that outcomes were better for reconstruction of these structures than for repair. Mariani et al. [27] found that there was a greater loss of flexion, and greater PCL instability, and a lower rate of return to preinjury activity levels in the primary repair group than in the reconstruction group. However, this study was limited by its small sample size (23 patients). In a retrospective review of 28 knees treated with primary repair of all damaged ligaments, Owens et al. [13] reported that patients had a good functional score and 92 % of the 25 patients were able to return to their previous jobs. Furthermore, a recent meta-analysis of 200 knees found that, compared with reconstruction, suture repair of the cruciate ligaments could achieve good clinical results and could serve as a treatment option for MLIK [20]. In the present study, we retrospectively analyzed 17 consecutive patients (18 knees) with in situ suture repair and found that the functional results (mean Lysholm score = 87.5) were comparable with those reported by Owens et al. (mean Lysholm score = 89) [13], Harner et al. (mean Lysholm = 87) [25], and Talbot et al. (mean Lysholm = 72) [32], Wascher et al. [33] (mean Lysholm = 88) [10], Yeh et al.(meanLysholm = 84) [34], and Liow et al. (mean Lysholm = 79) [24]. Furthermore, ligament reconstruction of injured ligaments is not recommended within the first days after injury due to the possible development of compartment syndrome [35], and early ligament reconstruction was considered to be an additional risk factor for arthrofibrosis [36]. Although there are no studies reported the relationship between arthrofibrosis and primary repair, delay ligament reconstruction is prone to arthrofibrosis as described above. Moreover, compared with ligament reconstruction, ligament repair could avoid the patient suffered the pain of autologous tendon or afforded the expense of artificial tendon.

Good knee range of motion and stability are important goals of treatment. Nohmi et al. [37] reported that double-bundle reconstruction could prove more stable throughout knee range of motion than single-bundle reconstruction. To increase the strength of the suture, we used a special suture technique—baseball running cross-stitch—with 2-0 COBRAID (Smith & Nephew) nonabsorbable suture to repair the injured ligaments in situ. The suture method differs from the Palmer suture [38] and the multiple-loop suture [39], both of which require joint immobilization for 6 weeks before rehabilitation can begin. The baseball running cross-stitch technique is more stable and not easy to avulse. Moreover, the PLC and MCL were also anchored in combination with the cross-stitch technique. Because this method strengthens the injured ligaments and increases immediate stability, most of the injured knees in our study had good range of motion.

The present study had some limitations. Because of the rarity of this injury, the number of patients was small. In addition, this was a retrospective review of a consecutive series of patients treated surgically with a unique technique, and there was no control group. Nevertheless, our study demonstrated good functional results of single-stage in situ suture repair of MLIK.

## Conclusions

Single-stage in situ suture repair of MLIK, using a baseball running cross stitch to repair all injured ligaments, could provide satisfactory subjective functional results, range of motion, and stability in the majority of the patients in this study. However, the procedure must be followed by a consistent postoperative rehabilitation program in early stage. Thence, single-stage in situ suture repair of MLIK is an effective treatment option. It could be considered as an alternate and effective option in the dislocation knee with multiple-ligament injury.

## Abbreviations

MLIK: Multipe-ligament injured knee
ACL: Anterior cruciate ligament
PCL: Posterior cruciate ligament
PLC: Posterolateral corner
MCL: Medial collateral ligament
VAS: Visual analogue score
SF-36: Health survey short form
DVT: Deep vein thrombosis

## References

[1] Levy BA, Fanelli GC, Whelan DB, Stannard JP, MacDonald PA, Boyd JL (2009). Controversies in the treatment of knee dislocations and multiligament reconstruction. J Am Acad Orthop Surg.

[2] Brautigan B, Johnson DL (2000). The epidemiology of knee dislocations. Clin Sports Med.

[3] KENNEDY JC (1963). Complete dislocation of the knee joint. J Bone Joint Surg Am.

[4] Levy BA, Dajani KA, Whelan DB, Stannard JP, Fanelli GC, Stuart MJ (2009). Decision making in the multiligament-injured knee: an evidence-based systematic review. Arthroscopy.

[5] Arom GA, Yeranosian MG, Petrigliano FA, Terrell RD, McAllister DR (2014). The changing demographics of knee dislocation: a retrospective database review. Clin Orthop Relat Res.

[6] Fanelli GC, Orcutt DR, Edson CJ (2005). The multiple-ligament injured knee: evaluation, treatment, and results. Arthroscopy.

[7] Kannus P, Jarvinen M (1990). Nonoperative treatment of acute knee ligament injuries. A review with special reference to indications and methods. Sports Med.

[8] Lobenhoffer P (2002). Complex instability of the anterior knee. Orthopade.

[9] Zhang Y, Zhang X, Hao Y, Zhang Y, Wang M, Zhou Y (2013). Surgical management of the multiple-ligament injured knee: a case series from Chongqing, China and review of published reports. Orthop Surg.

[10] Dedmond BT, Almekinders LC (2001). Operative versus nonoperative treatment of knee dislocations: a meta-analysis. Am J Knee Surg.

[11] Richter M, Bosch U, Wippermann B, Hofmann A, Krettek C (2002). Comparison of surgical repair or reconstruction of the cruciate ligaments versus nonsurgical treatment in patients with traumatic knee dislocations. Am J Sports Med.

[12] Stannard JP, Brown SL, Farris RC, McGwin GJ, Volgas DA (2005). The posterolateral corner of the knee: repair versus reconstruction. Am J Sports Med.

[13] Owens BD, Neault M, Benson E, Busconi B (2007). Primary repair of knee dislocations: results in 25 patients (28 knees) at a mean follow-up of four years. J Orthop Trauma.

[14] Hirschmann MT, Zimmermann N, Rychen T, Candrian C, Hudetz D, Lorez LG (2010). Clinical and radiological outcomes after management of traumatic knee dislocation by open single stage complete reconstruction/repair. BMC Musculoskelet Disord.

[15] Chhabra A, Cha PS, Rihn JA, Cole B, Bennett CH, Waltrip RL (2005). Surgical management of knee dislocations. Surgical technique. J Bone Joint Surg Am.

[16] Lysholm J, Gillquist J (1982). Evaluation of knee ligament surgery results with special emphasis on use of a scoring scale. Am J Sports Med.

[17] Tegner Y, Lysholm J (1985). Rating systems in the evaluation of knee ligament injuries. Clin Orthop Relat Res.

[18] Meyers MH, Harvey JJ (1971). Traumatic dislocation of the knee joint. A study of eighteen cases. J Bone Joint Surg Am.

[19] Meyers MH, Moore TM, Harvey JJ (1975). Traumatic dislocation of the knee joint. J Bone Joint Surg Am.

[20] Frosch K, Preiss A, Heider S, Stengel D, Wohlmuth P, Hoffmann MF (2013). Primary ligament sutures as a treatment option of knee dislocations: a meta-analysis. Knee Surg Sports Traumatol Arthrosc.

[21] Fanelli GC, Edson CJ (2002). Arthroscopically assisted combined anterior and posterior cruciate ligament reconstruction in the multiple ligament injured knee: 2- to 10-year follow-up. Arthroscopy.

[22] Fanelli GC, Edson CJ, Orcutt DR, Harris JD, Zijerdi D (2005). Treatment of combined anterior cruciate-posterior cruciate ligament-medial-lateral side knee injuries. J Knee Surg.

[23] Fanelli GC, Giannotti BF, Edson CJ (1996). Arthroscopically assisted combined anterior and posterior cruciate ligament reconstruction. Arthroscopy.

[24] Liow RY, McNicholas MJ, Keating JF, Nutton RW (2003). Ligament repair and reconstruction in traumatic dislocation of the knee. J Bone Joint Surg (Br).

[25] Richter M, Lobenhoffer P, Tscherne H. Knee Dislocation.Long-term results after operative treatment.Chirurg. 1999;70(11):1294–1301. 10.1007/s001040050782 10591767

[26] Rihn JA, Groff YJ, Harner CD, Cha PS (2004). The acutely dislocated knee: evaluation and management. J Am Acad Orthop Surg.

[27] Mariani PP, Santoriello P, Iannone S, Condello V, Adriani E (1999). Comparison of surgical treatments for knee dislocation. Am J Knee Surg.

[28] Bin SI, Nam TS (2007). Surgical outcome of 2-stage management of multiple knee ligament injuries after knee dislocation. Arthroscopy.

[29] Yastrebov O, Lobenhoffer P (2009). Treatment of isolated and multiple ligament injuries of the knee: anatomy, biomechanics, diagnosis, indications for repair, surgery. Orthopade.

[30] Fanelli GC, Edson CJ (2004). Combined posterior cruciate ligament-posterolateral reconstructions with Achilles tendon allograft and biceps femoris tendon tenodesis: 2- to 10-year follow-up. Arthroscopy.

[31] Harner CD, Waltrip RL, Bennett CH, Francis KA, Cole B, Irrgang JJ (2004). Surgical management of knee dislocations. J Bone Joint Surg Am.

[32] Talbot LA, Gaines JM, Huynh TN, Metter EJ (2003). A home-based pedometer-driven walking program to increase physical activity in older adults with osteoarthritis of the knee: a preliminary study. J Am Geriatr Soc.

[33] Wascher DC, Becker JR, Dexter JG, Blevins FT (1999). Reconstruction of the anterior and posterior cruciate ligaments after knee dislocation. Results using fresh-frozen nonirradiated allografts. Am J Sports Med.

[34] Yeh WL, Tu YK, Su JY, Hsu RW (1999). Knee dislocation: treatment of high-velocity knee dislocation. J Trauma.

[35] Peek RD, Haynes DW (1984). Compartment syndrome as a complication of arthroscopy. A case report and a study of interstitial pressures. Am J Sports Med.

[36] Balcarek P, Sawallich T, Walde TA, Ferlemann KG (2008). WachowskiM, Stu¨rmer KM, et al. Influence of cyclops syndromeafter anterior cruciate ligament reconstruction on thefunctional outcome. Sportverletz Sportschaden.

[37] Nohmi S, Ishibashi Y, Tsuda E, Yamamoto Y, Tsukada H, Toh S (2012). Biomechanical comparison between single-bundle and double-bundle anterior cruciate ligament reconstruction with hamstring tendon under cyclic loading condition. Sports Med Arthrosc Rehabil Ther Technol.

[38] Palmer I (2007). On the injuries to the ligaments of the knee joint: a clinical study. Clin Orthop Relat Res.

[39] Marshall JL, Warren RF, Wickiewicz TL, Reider B (1979). The anterior cruciate ligament: a technique of repair and reconstruction. Clin Orthop Relat Res.

